# *XRCC4*-related microcephalic primordial dwarfism: description of a clinical series of 7 cases, phenotype expansion and new diagnostic approaches

**DOI:** 10.1038/s41431-025-01821-0

**Published:** 2025-03-20

**Authors:** Silvestre Cuinat, Nicolas Chatron, Florence Petit, Perrine Brunelle, Etienne Dincuff, Marion Aubert Mucca, Eric Bieth, Ariane Schmetz, Harald Rieder, Bernd Wollnik, Silke Kaulfuß, Gökhan Yigit, Colina McKeown, Tim Savage, Meghan R. Mulligan, Louise S. Bicknell, Nicole Corsten-Janssen, Patrick Edery, Gaetan Lesca, Jean-Pierre de Villartay, Audrey Putoux

**Affiliations:** 1 Hospices Civils de Lyon, Service de Génétique, Centre Labélisé Anomalies du Développement CLAD Sud-Est, Lyon, France; 2 Centre de Recherche en Neurosciences de Lyon, équipe GENDEV, INSERM U1028 CNRS UMR5292, Université Claude Bernard Lyon 1, Lyon, France; 3 Institut Neuromyogène, Laboratoire Physiopathologie et Génétique du Neurone et du Muscle, Equipe Métabolisme énergétique et développement neuronal, CNRS UMR 5310, INSERM U1217, Université Claude Bernard Lyon 1, Lyon, France; 4 Univ. Lille, CHU Lille, ULR 7364 - RADEME, F-59000 Lille, France; 5 Laboratory « Genome Dynamics in the Immune System », INSERM UMR 1163, DGSI, Equipe labellisée La Ligue Nationale Contre le Cancer, Institut Imagine, Université Paris Descartes Sorbonne Paris Cité, 75015 Paris, France; 6 Service de Génétique Médicale, Hôpital Purpan, CHU, 31059 Toulouse, France; 7 Institute of Human Genetics, Medical Faculty and University Hospital Düsseldorf, Heinrich Heine University, Düsseldorf, Germany; 8 Institute of Human Genetics, University Medical Center Göttingen, Göttingen, Germany; 9 DZHK (German Centre for Cardiovascular Research), Partner Site Lower Saxony, Gottingen, Germany; 10 University of Göttingen, Cluster of Excellence “Multiscale Bioimaging: from Molecular Machines to Networks of Excitable Cells” (MBExC), Göttingen, Deutschland; 11 Genetic Health Service, Wellington Children’s Hospital, Wellington, New Zealand; 12 General Pediatrics, Diabetes and Endocrinology, Wellington Children’s Hospital, Wellington, New Zealand; 13 Department of Biochemistry, University of Otago, Dunedin, New Zealand; 14 Department of Genetics, University of Groningen, University Medical Center Groningen, Groningen, The Netherlands

**Keywords:** Neurodevelopmental disorders, Endocrine system and metabolic diseases, Immunological disorders

## Abstract

The non-homologous end joining (NHEJ) pathway is essential to repair DNA double-strand breaks. XRCC4 acts as a stabilizer of the DNA ligase LIG4 in the NHEJ process. In humans, *XRCC4* pathogenic variants are responsible for a microcephalic primordial dwarfism syndrome (MPD). Currently, 17 patients have been reported with *XRCC4*-related MPD and we report 7 new patients from 6 different families, including one fetus. The patients present with short stature, severe microcephaly, neurodevelopmental disorder and additional features, such as transient increase in nuchal translucency, congenital glaucoma, thumb anomalies, hepatic steatosis, seizures, essential tremor and oligodontia which have not been previously described. Hyper- and hypopigmented skin macules, dermatofibrosarcoma, mandibular osteoid osteoma and pancytopenia are also new features, reminiscent of cancer susceptibility syndromes. Functional studies were performed on two patients carrying the known pathogenic p.(Trp43Arg) variant in homozygous state, using a fast, cost-effective and non-invasive approach on PBMCs: (1) Survival analyses after ionizing radiation confirm important radiosensitivity. (2) Flow cytometry showed the lack of TCR-Va7+ T-lymphocytes, suggesting recombination defect of V(D)J coding segments. (3) This was confirmed by multiplexed RT-PCR (PROMIDISα biomarker), analyzing the diversity of V(D)J coding segments in a subset of the TCRα repertoire. We therefore extend the phenotype of *XRCC4*-related MPD and suggest a combination of three functional assays, based on radiosensitivity and V(D)J recombination defect, to improve the interpretation of *XRCC4* variants in fast, cost-effective and non-invasive manner. These findings will improve the diagnosis, genetic counselling, follow-up and management of these patients.

## Introduction

Double-strand breaks (DSB) of DNA, caused by radiation, chemical agents or replication accidents [1], can be repaired through two main systems. Ideally, the Homology Directed Repair (HDR) system, accurate and precise, acts when strands are close together, for example during the early stages of mitosis. Alternatively, but more frequently, the Non-Homologous End Joining (NHEJ) repair system, erratic and inaccurate, repairs DSB by direct DNA end joining when there is no homologous template nearby [2].

The NHEJ system operates as follows: free DNA ends are bound by the Ku70-Ku80 heterodimer, which recruits the DNA-dependant protein kinase DNAPK (encoded by the *PRKDC* gene) and the nuclease Artemis (encoded by the *DCLRE1C* gene). Artemis processes damaged DNA ends when required, then polymerases μ and λ synthesize new extremities. The final ligation is performed by the DNA ligase LIG4, stabilized by XLF, XRCC4 and its paralogue PAXX [2].

Of note, the NHEJ system is also involved in the immune system in a tissue-specialized manner, playing an essential role in the somatic recombination process of the variable (V), diversity (D), and joining (J) segments. These V(D)J coding gene segments undergo programmed double-strand cleavage at the recombination signal sites by RAG1 and RAG2. Then the NHEJ components introduce random deletions and insertions before final ligation by the XRCC4-XLF-LIG4 complex. This ensures the diversification of the T-cell receptor (TCR) and immunoglobulin repertoires in T and B lymphocytes, respectively [3].

The NHEJ system is therefore essential in adaptive immunity, in protection against ionizing stress and tumorigenesis. Moreover, descriptions of cognitive impairment in patients with pathogenic variations in *PRKDC* [4] and in the three genes of the NHEJ ligation complex, *NHEJ1/XLF* [5], *LIG4* [6] and *XRCC4* [7] also revealed an essential role for the NHEJ complex in neurodevelopment [8].

Shaheen et al. first reported a *XRCC4* (MIM*194363) homozygous variants in a patient presenting with microcephalic primordial dwarfism (MPD; MIM#616541) [9]. Since then, 17 patients were reported in 12 different families [7, 9–14]. The patients show extreme growth failure and microcephaly, varying degrees of developmental delay ranging from normal cognition to severe intellectual disability, urogenital malformations, dilated cardiomyopathy and endocrine dysfunction including type 2 diabetes or glucose intolerance, dyslipidemia, hypergonadotropic hypogonadism, hypothyroidism and multinodular goiter. Craniofacial abnormalities include triangular face, sloping forehead, deep-set eyes, prominent beaked nose and pointed chin. Surprisingly for NHEJ deficiency, these patients do not present with obvious immune deficiency [15].

Here, we describe 7 new patients with additional clinical features expanding the phenotype of *XRCC4*-related microcephalic primordial dwarfism (*XRCC4*-related MPD), and propose a combination of three functional assays to reclassify variants of uncertain significance (VUS) in *XRCC4*, in a fast, cost-effective and non-invasive way.

## Material and methods

### Data collection

Two siblings were diagnosed with *XRCC4*-related MPD by exome sequencing (ES) in Lyon university medical center. A call for collaboration was made through the French network AnDDI-Rares (developmental abnormalities and malformative syndromes), and the European Rare disease network ERN-ITHACA. The aim was to collect clinical and molecular data from patients with *XRCC4* pathogenic variants, in order to better describe and diagnose this rare syndrome. We collected clinical and molecular data on 7 new patients from 6 unrelated families. All families were evaluated by a clinical geneticist.

### Genetic testing

Written consent for genetic testing was obtained for each patient. DNA was extracted from blood samples for all patients and their parents. ES and gene panel were performed following protocols described in Supplementary Data. Variant interpretation and classification were done according to American College of Medical Genetics guidelines. *XRCC4* variants are annotated according to the NM_003401 reference transcript. The bi-parental inheritance of *XRCC4* pathogenic variants was verified for each patient by Sanger sequencing in their parents, with the exception of P4 due to the unavailability of parental blood samples.

### Functional studies

Three tests were carried out on PBMCs, isolated from heparinized blood of siblings P1 and P2, carrying the known pathogenic p.(Trp43Arg) variant in homozygous state [7], and of their healthy heterozygous parents. Given the small blood sample, functional tests were carried out once in each individual, with concordant results.

(i) *Cellular sensitivity to ionizing radiation*. PBMCs were subjected to increasing doses of ionizing radiations. The test was performed in duplicate for each dose. After resting 4 h in complete culture medium (RPMI 10% FCS) to allow for DNA repair, T lymphocytes were activated with CD3/CD28 coated beads (Dynabeads) and cultured in the presence of IL2 (10^3 ^u/ml) in 48-well plates. After 6 days of culture, cells were recovered, washed, and counted through flow cytometry. The survival fraction represents the cell count of irradiated cells relative to untreated cells. Data were compared to the mean survival of PBMCs obtained from 17 healthy controls, 31 patients with ataxia telangiectasia carrying mutations in ATM, and 6 patients with DNA Lig4 mutations. The activation/proliferation of PBMC in the absence of irradiation was compared between the various samples and a healthy control (Supplementary Fig. 1).
(ii) *TCR-Vα7 dosage*. After red cell lysis and wash, whole blood was incubated with a cocktail of fluorescently labelled antibodies as described previously [16]: APC-Cy7 anti-CD3, PE anti-TCR-Va7.2 (Sony), and PE-Vio770 anti-CD161 (Milteny). The proportion of CD3 + /CD161-/Vα7.2+ was determined by flow cytometry analysis. In this analysis, gating on CD161-/Va7.2 + T lymphocytes allows the exclusion of CD161+ clonal MAIT cells (Mucosal-Associated Invariant T-cells) which express an invariant TCR-Vα7 positive TCR, and the proportion of which can be highly variable depending on health status, causing a potential bias.
(iii) *PROMIDISα*. The PROMIDISα signature was determined from whole PBMCs as previously described [16] and validated in several NHEJ-related disorders [17]. Briefly, RNA was purified from PBMC and reverse transcribed. A subset of TCR repertoire was then amplified by multiplex PCR using 8 forward Vα primers representing proximal (TRAV35, TRAV41), middle (TRAV20, TRAV21, TRAV23), and distal (TRAV1, TRAV5, TRAV10) Vα segments and a constant region (Cα) specific reverse primer. PCR products were further processed for NGS Illumina sequencing. TCR-Vα and Jα usage was assigned through IMGT/HighV-Quest [https://www.imgt.org/HighV-QUEST/home.action] [18] and quantified using custom R script. PROMIDISα signature was evaluated through hierarchical clustering using FactoMineR [19] against our in-house PROMIDISα database containing signatures for 24 healthy controls, 18 patients with various defects in V(D)J recombination and/or DNA repair (5 *DCLRE1C*, 3 *LIG4*, 4 *Cernunnos/XLF*, 2 *PRKDC*, 4 *RAG1* patients), and 34 ataxia telangiectasia patients with *ATM* gene variants.

## Results

### Patients and families

**Fig. 1:**
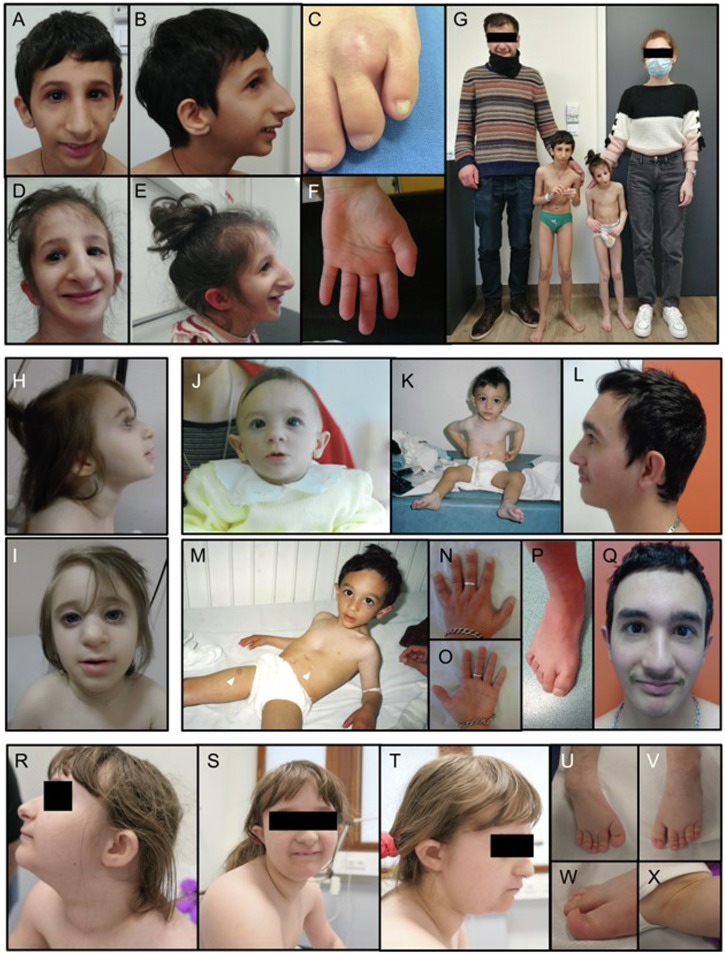
Photographs of patients with *XRCC4*-related MPD. **A**, **B** P1 at 12 years 5 months. Note sloping forehead, deep set eyes, low-set, posteriorly rotated ears, beaked nose with sharp nasal root and broad tip, low columella with short philtrum, pointed chin giving a triangular face shape. **C** Dermatofibrosarcoma of the right foot in P1 at the age of 6, before surgical resection. **D**–**F** P2 at 11 years 3 months. Note facial dysmorphia similar to her brother P1 (**D**, **E**), broad thumbs with distal implantation (**F**). **G** P1 and P2 with their parents. **H**, **I** P3 at 4 years. Note the slightly beaked appearance of the nose, with a broad bulbous tip, and a thin upper lip. **J**–**Q** P4 at 6 months (**J**), 18 months (**K**), 3 years (**M**) and 22 years (**L**, **N**–**Q**). Note the hyper- and hypochromic macules (**M**, arrows), and the short broad appearance of the fingers (**N**, **O**), with a single left transverse palmar crease (**O**). On the feet, note partial syndactyly 2-3 with fourth/fifth brachymetatarsia (**P**). **R**–**X** P5 at 11 years and 3 months. Note the low posteriorly rotated ears with abnormal helix, a broad nose with bulbous and broad nasal tip with low columella, a short philtrum and a thin upper lip (**R**–**T**). Note also the presence of acanthosis nigricans on the neck (**R**) and the axilla (**X**). On the feet, a mild fourth/fifth brachymetatarsia is observed, with bilateral equinovarus (**U**, **V**) and nails deformities (**W**).

We identified 7 new patients with *XRCC4*-related MPD, 4 males and 3 females from 6 different families, including one fetus. Clinical data are summarized in Table 1 and Fig. 1. Detailed medical history is provided for each patient in Supplementary Results and Table S1. Patients were aged from 14 WG to 22 years at last examination. All presented with pre- and postnatal growth retardation, microcephaly and intellectual disability when assessed. Genitourinary anomalies, glucose intolerance, dyslipidemia and hematological abnormalities were found in half of patients. Three patients had lymphopenia, which was progressive in one. Additional features are discussed below.

**Table 1:** Clinical features of patients with *XRCC4*-related MPD, in our series and in addition to patients in the literature.

	Present cases	%	Total cases	%	Details
**Patients**	7		24		
**Families**	6		18		
**Sex**	3F/4M		8F/16M		
**Antenatal, Birth, Neonatal**					
IUGR	(7/7)	100	(21/21)	100	
Other antenatal abnormality	(3/7)	43	(6/24)	25	(a)
Premature delivery	(3/6)	50	(7/16)	47	
Birth weight: mean SD (min; max)	−2,7 (−2; −4)		−3 (−1,3 ; −5,3)		
Birth length: mean SD (min; max)	−3,7 (−0,5; −7,5)		−3,8 (−0,5; −7,5)		
Birth OFC: mean SD (min; max)	−3,6 (−2,2; −4,5)		−3,6 (−1,8; −4,8)		
**Growth**					
Short stature	(6/6)	100	(22/22)	100	
Microcephaly	(6/6)	100	(20/20)	100	
Weight at last assessment: mean SD (min; max)	−5,1 (−3,8; −7)		−5,2 (−3,8; −7)		
Height at last assessment: mean SD (min; max)	−6,0 (−3,7; −7)		−5,1 (−1,8; −7,2)		
OFC at last assessment: mean SD (min; max)	−8,2 (−4,4; −10,3)		−7,3 (−2,9; −11)		
**Neurodevelopment**					
Developmental delay	(5/6)	83.33	(14/17)	82	
Intellectual disability	(5/5)	100	(12/14)	86	(b)
Motor developmental delay	(3/6)	50	(3/9)	33	
Speech delay	(5/6)	83.33	(8/9)	89	
**Neurological abnormalities**					
Tremor	(2/6)	33.33	(2/23)	9	
Ataxia	(0/6)	0	(3/23)	13	
Sensory neuropathy	(0/6)	0	(3/23)	13	
Pyramidal sign	(2/6)	33.33	(5/23)	22	
Muscle weakness	(0/6)	0	(2/23)	9	
Cognitive deterioration	(0/6)	0	(2/23)	9	
Seizures	(1/6)	16.67	(1/23)	4	
Brain MRI abnormalities	(1/6)	16.67	(5/24)	21	(c)
**Sensorial abnormalities**					
Hearing impairment / Deafness	(0/6)	0	(1/23)	4	(d)
Visual impairment	(2/6)	33.33	(5/23)	22	(e)
**Craniofacial abnormalities**					
Triangular face	(4/6)	66.67	(11/19)	58	
Slopping forehead	(3/6)	50	(6/19)	32	
Low set ears	(6/6)	100	(6/19)	32	
Hypotelorism	(0/6)	0	(4/19)	21	
Deep-set eyes	(4/6)	66.67	(9/19)	47	
Prominent beaked nose	(4/6)	66.67	(9/19)	47	
Low columella	(4/6)	66.67	(9/19)	47	
Short philtrum	(4/6)	66.67	(7/19)	37	
Dental abnormalities	(2/6)	33.33	(3/19)	16	(f)
Pointed chin	(4/6)	66.67	(9/19)	47	
**Skeletal abnormalities**					
Fifth finger clinodactyly	(2/6)	33.33	(5/23)	22	
Delayed bone age	(2/6)	33.33	(3/23)	13	
Syndactyly of 2–3 toes	(1/6)	16.67	(2/23)	9	
Short 4th and 5th metatarsals	(3/6)	50	(3/23)	13	
Broad thumbs	(1/6)	16.67	(1/23)	4	
Equinovarus feet	(1/6)	16.67	(1/23)	4	
Pes cavus	(0/6)	0	(2/23)	9	
**Visceral abnormalities**					
Cardiac	(0/6)	0	(3/23)	13	(g)
Renal	(1/6)	16	(3/23)	13	(h)
Genital					
*Male*	(2/3)	66	(7/15)	47	(i)
*Female*	(1/3)	33	(1/8)	13	(j)
Gastrointestinal	(1/6)	16	(2/23)	9	(k)
**Dermatological abnormalities**					
Acanthosis nigricans	(1/6)	16	(3/23)	13	
Pigmented and depigmented spots	(1/6)	16	(1/23)	4	
Eczema	(0/6)	0	(1/23)	4	
Hands hyperhydrosis	(1/6)	16	(1/23)	4	
**Hematological abnormalities**					
Anemia	(1/6)	16	(3/23)	13	
Lymphopenia	(3/6)	50	(6/23)	26	
Neutropenia	(1/6)	16	(3/23)	13	
Thrombocytopenia	(1/6)	16	(3/23)	13	
Pancytopenia	(1/6)	16	(1/23)	4	
Abnormal Ig profile	(1/6)	16	(1/23)	4	
**Metabolic and endocrine dysfunction**					
Type 2 diabetes/glucose intolerance	(3/6)	50	(8/23)	35	
Dyslipidemia	(3/6)	50	(7/23)	30	
Hypergonadotropic hypogonadism	(2/6)	33	(6/23)	26	
Hypothyroidism	(0/6)	0	(3/23)	13	
Multinodular goiter	(0/6)	0	(2/23)	9	
Hepatic steatosis	(1/6)	16	(1/23)	4	
**Other**					
Feeding difficulties	(3/6)	50	(5/23)	22	(l)
History of cancer or neoplasia	(2/6)	33	(4/23)	17	(m)
Defective thermoregulation	(1/6)	16	(1/23)	4	

*IUGR* Intra-Uterine Growth Restriction, *SD* c, *OFC* Occipital-Frontal head Circumference, *MRI* Magnetic Resonance Imaging

(a) oligohydramnios 2/22, ventriculomegaly 1/22, unilateral renal hypoplasia 1/22, transient increase in nuchal translucency 1/22; (b) mild 5/8, moderate 2/8, severe 1/8; (c) ventriculomegaly 2/22, vermis atrophy 2/22, thin corpus callosum 1/22, small caudate nuclei 1/22, simplified gyral pattern 1/22, pituitary hypoplasia 1/22; (d) moderate hearing loss 1/22; (e) bilateral cataract 1/22, bilateral glaucoma 1/22, strabismus 1/22, astigmatism 1/22, hyperopia 2/22; (f) oligodontia 1/18, microdontia 1/18, malpositioned teeth 1/18; (g) dilated cardiomyopathy 3/3; (h) hypoplasia 2/22, ectopic kidney 1/22, unilateral agenesis 1/22; (i) cryptorchidism 7/15, micropenis 2/15; (j) hypoplastic labia minor 1/7; (k) severe gastroesophageal reflux 1/22, inguinal hernia 1/22; (l) requiring gastrostomy in 4/5 cases; (m) dermatofibrosarcoma 1/4, mandibular osteoid osteoma 1/4, thalamic glioma 1/4, jejunal GIST 1/4.

### Genetic results

**Fig. 2:**
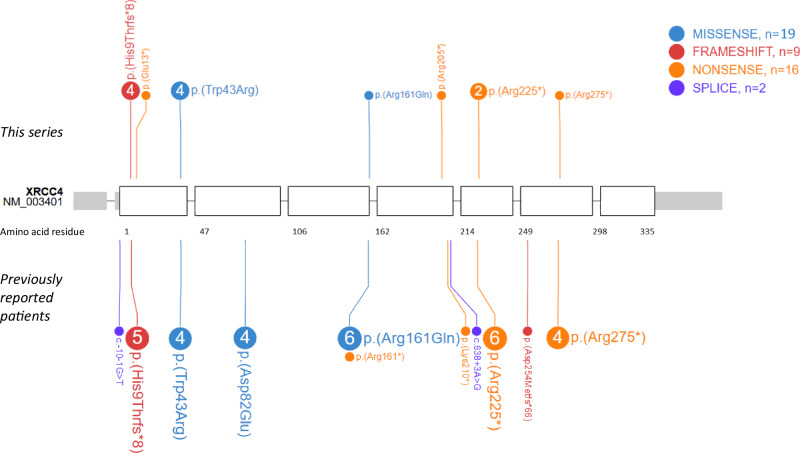
Pathogenic *XRCC4* variants in patients reported here and previously. The schematic view of *XRCC4* exons (NM_003401) and the amino-acid numbering are based on PeCan Data of Proteinpaint.

The patients in our series were diagnosed by ES (6/7) or NGS gene panel (1/7). Age at diagnosis was highly variable, ranging from the prenatal period at 15 WG (P7) to adulthood (P4). Biallelic pathogenic *XRCC4* variants, presented in Fig. 2, were found at homozygous state in patients P1-2, and at compound heterozygous state in patients P3-7. Among the 7 different variants including 2 missenses *[p.(Trp43* *Arg); p.(Arg161Gln)]*, 1 frameshift *[p.(His9ThrfsTer8)]* and 4 nonsense variants *[p.(Glu13Ter); p.(Arg205Ter); p.(Arg225Ter); p.(Arg275Ter)]*, only the p.(Glu13Ter) and p.(Arg205Ter) variants have not been previously reported.

### Functional investigations

**Fig. 3:**
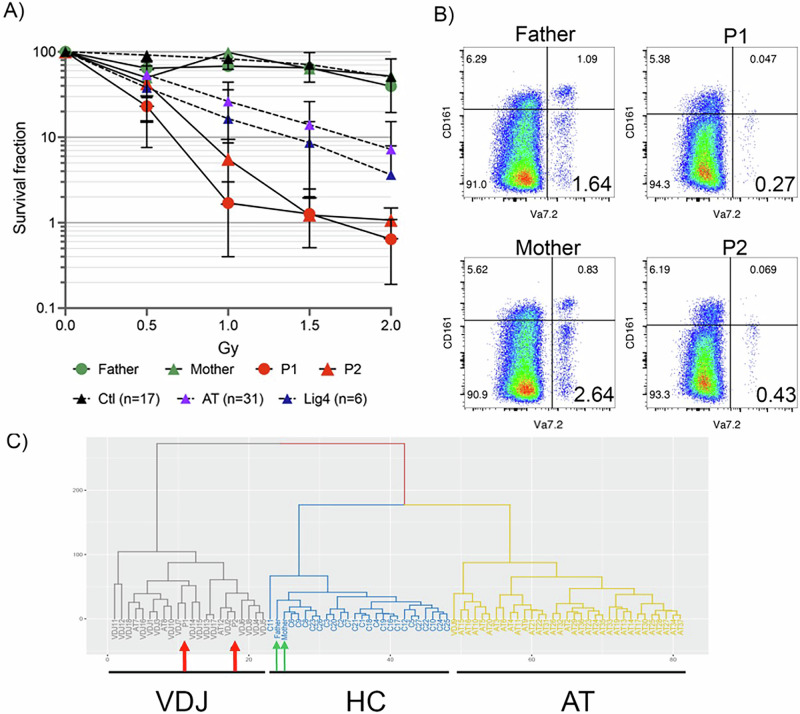
DNA repair biomarkers in PBMCs. **A** Cellular sensitivity of peripheral T lymphocytes to increasing doses of ionizing radiations in patient 1 (P1), patient 2 (P2) and parents. Mean IR sensitivity of cells from 17 healthy controls (Ctl), 6 patients with *LIG4* mutations, and 31 patients with ATM mutations are included for comparison. **B** Quantification of CD161 negative T lymphocytes expressing TCR-Vα7.2 through flow cytometry. **C** Hierarchical clustering of PROMIDISα signatures by principal component analysis (PCA). VDJ: patients with VDJ/DNA repair deficiency (*n* = 18, including 5 *DCLRE1C*, 3 *LIG4*, 4 *Cernunnos/XLF*, 2 *PRKDC*, 4 *RAG1*), HC: Healthy controls (*n* = 24), AT: Ataxia telangiectasia patients (*n* = 34). P1 and P2: red arrows. Mother and father: green arrows.

Functional investigations have been performed on extracted cells from blood samples of P1 and P2. Cellular sensitivity to increasing doses of ionizing radiations (IR), causing DNA breaks, was evaluated on blood derived primary T-lymphocytes. The T cell activation/proliferation of P1 and P2 was slightly (2-fold) reduced as compared to both parents and a healthy control (Supplementary Fig. 1). In contrast, as shown in Fig. 3A, T-cells from both patients showed an important decrease in proliferation upon IR (up to 2 log-fold reduction at 1.5 Gy) as compared with that of cells from 17 healthy controls (HC), attesting a major defect in the DNA damage response. The increased sensitivity of P1 and P2 was even stronger than that of several patients with mutations in either ATM or Lig4 genes. IR sensitivity of cells from both heterozygous parents were comparable to HC. The DNA repair defect of P1 and P2 translated in typical signatures using the two previously described TCR repertoire-based biomarkers [16]. First the proportion of CD3 + /CD161- non-MAIT T-lymphocytes expressing TCR-Vα7.2 was strongly reduced in both patients (0.27% and 0.43%, respectively) as compared to the normal values in both parents (1.64% and 2.64% respectively) (Fig. 3B). Moreover, after multiplexed PCR and NGS sequencing to analyze a subset of the TCRα repertoire, principal component analysis revealed a PROMIDISα signature typical of VDJ/DNA repair defective conditions for both patients, characterized by a bias in T-cell repertoire towards T-lymphocytes expressing proximal Vα and Jα segments (Fig. 3C). Both parents presented a PROMIDISα signature that clustered with the group of healthy controls.

## Discussion

### Clinical spectrum of XRCC4-related MPD

**Fig. 4:**
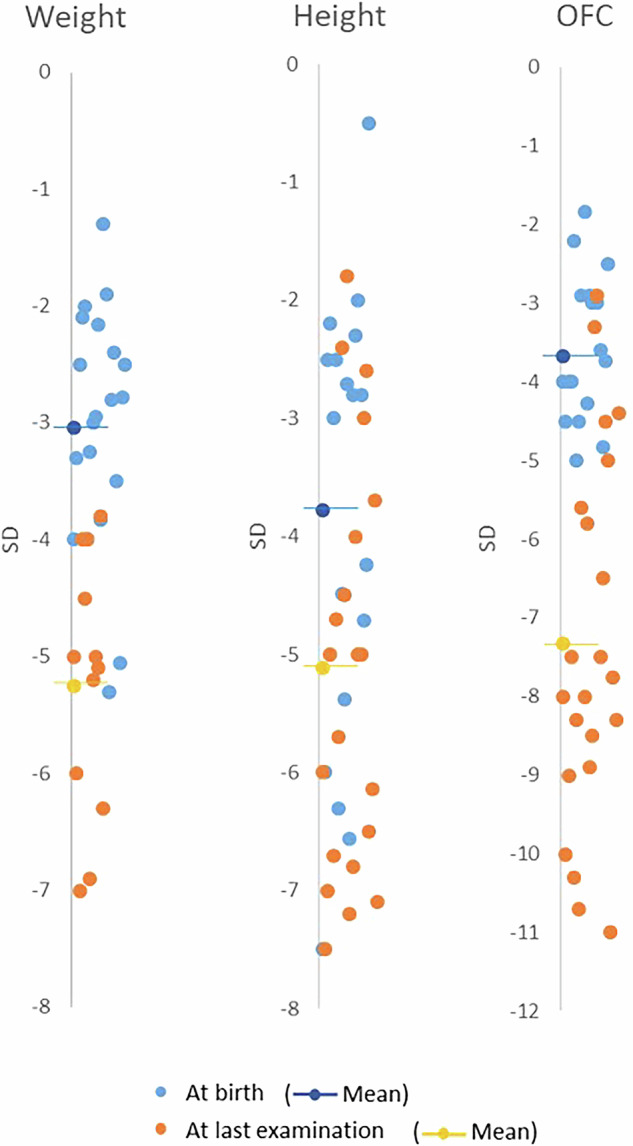
Measurements of patients with *XRCC4*-related MPD. For each patient newly or previously reported, weight, height and OFC are represented in standard deviations compared with WHO standards, at birth (blue spots) and at last evaluation (orange spots). It should be noted that while the IUGR is present in most patients, there is also a postnatal worsening in growth retardation, with a predominant impact on the OFC. The means are also represented by a slashed dot.

17 *XRCC4*-related MPD patients from 12 families were previously reported [7, 10–14] and we report here 7 new patients from 6 different families, including one fetal case, the first antenatal diagnosis reported to our knowledge. We performed a literature review of the phenotype and genotype of all patients previously described, and reported here (Tables 1 and S1). All patients had a proportionate IUGR, sometimes as early as the first trimester of pregnancy, followed by severe postnatal growth retardation and microcephaly. Their height ranged from −0.5 to −7.5 SD at birth with relative stability in the postnatal period (−1.8 to −7 SD). In contrast, their OFC at birth ranged from −1.83 to −4.83 SD, and the microcephaly progressively worsened in the postnatal period, to become the most affected parameter (−2.9 to −11 SD) (Fig. 4). Some patients present the classical facial features described in some type of MPD like Seckel-syndrome, with a triangular shaped face, a slopping forehead, low set ears, deep-set eyes, a prominent beaked nose, a low columella with short philtrum, and micrognathia (Fig. 1A–I). However, other patients do not present these characteristics (Fig. 1J–X). Facial features changed with age in P4, becoming thicker and coarser in adulthood (Fig. 1J–Q), reminiscent of patient published by Guo et al. [12]. Most patients have developmental delay, particularly affecting language, and cognitive impairment of variable severity. Neurological abnormalities are sometimes observed, mostly in adulthood, including sensory neuropathy, progressive cerebellar and pyramidal syndrome, muscle weakness, and cognitive deterioration. We report new neurological features, namely seizures in 1 patient and essential tremor in 2 patients. Sensory involvement is also observed, with visual impairment in some patients due to cataract, bilateral glaucoma, strabismus, astigmatism, or hyperopia—while hearing loss appears to be rare. We confirm that feeding difficulties during infancy can be part of the syndrome and can be severe: in our series, 3 patients had such symptoms, and all of them required gastrostomy. Cardiac and renal involvement appear to be rare. Dilated cardiomyopathy has been reported in 3 cases, 2 of adult onset [10], and one from 2 months of age leading to early death [14], which could illustrate the wide variability in the expression of this disease, although a second event cannot be excluded in the context of consanguinity. Variation in genital development seems to be frequent in males (cryptorchidism in almost half of patients and micropenis in 2 patients). Metabolic and endocrine abnormalities appear to be frequent, namely type 2 diabetes or prediabetes, hypothyroidism, hypergonadotropic hypogonadism, dyslipidemia including hypercholesterolemia and hypertriglyceridemia. Dyslipidemia was severe and rapidly worsened in our patient P4 between 19 and 21 years of age, requiring triple treatment to limit hepatic steatosis and avoid acute pancreatitis. Finally, cytopenias are reported in almost half of patients, including lymphopenia. Although subclinical abnormalities in blood count have already been reported [7, 10, 11, 13], P3 was the first patient with severe pancytopenia, from which she probably died. Our findings also include significant immunodeficiency in P1 and P2 despite normal or subnormal blood count before immunophenotyping (Table S2). While the amount of polynuclear cells, monocytes, CD8 + T-lymphocytes and NK-cells, seems normal, we show in both patients a significantly low rate of B-lymphocytes and CD4 + T-lymphocytes, predominantly in the naive subpopulation (CD4 + CD45RA + ), with a CD4+ count below 500 cells/μL, theoretically compatible with opportunistic infections [20]. In addition to tremors and convulsions, we report new features that have not been previously described, namely transient increase in nuchal translucency, congenital glaucoma, thumb anomalies, hypoplastic labia minora, oligodontia, hepatic steatosis, café-au-lait and hypochromic macules and defective thermoregulation with multiple hypothermic episodes (see detailed medical history in Supplemental Results).

### Tumor susceptibility

Only two patients in the literature have been reported with tumors [11, 12], and we report two other cases with new types of tumors. So, four types of tumor have been now reported in patients with *XRCC4*-related MPD, namely dermatofibrosarcoma, mandibular osteoid osteoma, thalamic glioma, and jejunal gastrointestinal stromal tumor (GIST), respectively at 5, 20, 19 and 30-years-old. These tumors seem more differentiated and with a lower malignant grade than those in other DNA repair deficiencies (i.e sarcoma, leukemia, lymphoma or adenocarcinoma) [21]. Dermatofibrosarcoma protuberans is a low-grade malignant tumor with aggressive local infiltration, usually diagnosed between 30 and 50-years-old [22], and rarely observed in children [23]. Its association with tumor predisposing disorders is not classic, but it has exceptionally been reported in Li-Fraumeni syndrome [24] and interestingly, Fanconi anemia [25]. Osteoid osteomas are benign tumors, common before 30-years-old [26]. However, localization in the mandible is observed in less than 1% of cases [27] and may suggest Gardner tumor predisposing syndrome [MIM#175100]. GIST represents less than 1% of all gastrointestinal tumors and only 10% originate in the jejunum. 10–30% progress to malignancy [28]. Rarely, these tumors are associated with germline variants in *PDGFRA* (MIM*173490), *SDHB* (MIM*185470), *SDHC* (MIM*602413), and *KIT* (MIM*164920). Finally, thalamic gliomas represent less than 1% of brain tumors [29] and may exceptionally be associated with tumor predisposition syndromes such as Li-Fraumeni [MIM#151623], type-1 neurofibromatosis [MIM#162200], or Constitutional Mismatch Repair Deficiency Syndrome impacting the DNA mismatch repair system [MIM:PS276300]. Thus, although these tumors do not appear to be classically associated with any known tumor predisposition syndromes, their rarity, their atypical location and early onset make the hypothesis of a chance association with *XRCC4*-related MPD unlikely. Pancytopenia in P3, increased chromosomal breakage under mitomycin-C, café-au-lait and hypopigmented macules in P4, also reinforce the hypothesis of a tumor susceptibility in these patients, reminiscent of other DNA repair disorders such as Fanconi anemia (see below). Importantly, although the types of tumor observed does not make it possible to suggest organ-specific monitoring at the present time, the radiosensitivity in these patients prompts us to formally recommend limiting exposure to radiation, particularly in the context of medical care, where a large number of x-ray images tends to be performed in the context of short stature. Likewise, should a tumor develop in *XRCC4* patient, therapy must be extremely cautious to avoid the morbidity associated with the acute toxicity of genotoxic treatments, as previously documented in a *LIG4* patient [30].

### Differential diagnosis

The main differential diagnoses include other MPD, especially Seckel (MIM:PS210600), Majewski (MIM#210720) or Taybi-Linder syndromes (MIM#210710), due to severe growth retardation and overlapping facial features such as triangular face and prominent beaked nose. Like *XRCC4*, other DNA maintenance genes are involved in MPD, including *ATR* [MIM*601215], *TRAIP* [MIM*605958], *RBBP8* [MIM*604124], *RECQL3* [MIM*604610], *NBN* [MIM*602667], *DDX11* [MIM*601150], *ERCC6* [MIM*609413], *ERCC8* [MIM*609412], and *LIG4* [MIM*601837]. Other disorders involving the NHEJ system also can be considered as differential diagnoses, since pathogenic variations impacting *PRKDC* [MIM*600899], *NHEJ1* [MIM*611290], and *LIG4* [MIM*601837] can manifest as MPD with neurodevelopmental disorder, but in these syndromes, severe combined immunodeficiency (SCID) is at the forefront, in contrast to *XRCC4*-related MPD. More rarely, Fanconi anaemia can also manifest as MPD [9], suggesting a phenotypic continuum within these syndromes.

Other DNA repair deficiencies may also be reminiscent of *XRCC4*-related MPD owing to cancer susceptibility, cytopenia, hyper/hypopigmented macules, radial/thumb anomalies, neurodegenerescence and endocrine dysfunction. Indeed, tumors and cytopenia observed in some *XRCC4* patients also remind cancer susceptibility in *LIG4* deficiency [31], Fanconi anemia [MIM:PS227650], Ataxia-Teleangiectasia [MIM#208900], and more broadly, genomic instability syndromes such as Mosaic Variegated Aneuploidy [PS257300]. The co-occurrence of hypochromic and café-au-lait spots as described in P4 also reminds Fanconi anaemia, Bloom, or Nijmegen syndromes [32]. Likewise, radial/thumb anomalies observed in P2 are also reminiscent of Fanconi anaemia [MIM:PS227650] and *ATR*-related Seckel syndrome [MIM#210600], more rarely observed in *LIG4*-related MPD [31], Nijmegen [33] and Bloom syndromes [34]. Neurodegenerescence, reported in two adults with *XRCC4*-related MPD [10], and suspected in P1 based on progressive pyramidal signs and tremor, is a main feature in Ataxia-Teleangiectasia [MIM#208900], well-reported in Cockayne [MIM#133540, MIM#216400] and Nijmegen syndromes [MIM#251260]. Endocrine dysfunction, well reported in *XRCC4*-related MPD, is also observed in other DNA repair defects, including *DNA2*, *BLM*, *NSMCE2*-related MPD [MIM#615807, MIM#210900, MIM#617253], but also in other MPD such as Majewski syndrome [35]. Finally, the abnormal mitomycin-C chromosome breakage test observed on P4 also reinforces the overlap with Fanconi anaemia.

### XRCC4 pathogenic variants

In our series, the p.(Glu13Ter) and p.(Arg205Ter) variants (in P6 and P7 respectively) were not previously reported, but some variants appear to be recurrent (Fig. 2). The p.(His9ThrfsTer8) variant, in particular, was found in 9 patients from 8 different families. It is present in the heterozygous state 673 times in the control database gnomAD (v4.1.0), including 624 European individuals, strongly suggesting a founder effect. Likewise, the p.(Arg225Ter) and p.(Arg275Ter) variants are each found in 5 different families and are present respectively 32 and 10 times in the heterozygous state in European population. The p.(Trp43Arg) variant, homozygous in 4 patients from 3 families, is present 11 times in gnomAD, in the heterozygous state, exclusively in the South Asian population. This suggests a founder effect in this population, given that our patients P1 and P2 are known to originate from Georgia. No variant in our series is present in the homozygous state in gnomAD (v4.1.0).

Of the 24 patients now reported, 11 carry pathogenic variants in the homozygous state and 13 in the compound heterozygous state. The 13 different variants now reported are mainly truncating variants (8/13; nonsense or frameshift) but also missense (3/13) and splicing variants (2/13). Of note, no genotype-phenotype correlation emerges from these data, and patients with biallelic truncating variants in *XRCC4* do not appear to have a more severe phenotype (P4,5,6). Missense variants also have a loss-of-function effect, with aberrant splicing and synthesis of a truncated protein (p.Asp82Glu [11]; p.Arg161Gln [13]), or a reduction in protein stability (p.Trp43Arg) [7]. Likewise, the c.-10-1G > T variant induces aberrant splicing, leading to a major reduction in *XRCC4* protein level, even with a transcript that remains detectable [7]. These results all point to a biallelic loss-of-function of *XRCC4* as a common pathomechanism.

### Pathomechanisms

Most MPDs are caused by cell cycle abnormalities in progenitor cells, at early stages of embryogenesis [36]. Several MPDs are due to DNA repair defects, which represent an indirect mechanism of “Cell Cycle-opathies”, causing early apoptosis and cell cycle arrest [37]. Interestingly, residual LIG4 ligase activity remains detectable in *XRCC4* patients [7], and could explain viability in humans but not in complete KO mice [38]. Unlike *XRCC4*-patients, true biallelic KOs are less likely in patients with LIG4 deficiency, as this gene consists of a single coding exon. It is likely that truncating variations result in a truncated protein rather than mRNA degradation by NMD, explaining why a continuum of severity correlated with the position of the truncating variants is observed in LIG4 deficiency [31]. LIG4 is therefore partially destabilized by XRCC4 deficiency, but it has recently been shown that other factors (PAXX, ATM) may play a compensatory role in the stabilizing function of LIG4 in mice [39], and other *XRCC4* paralogs (including *PAXX, XLF*) could have the same role in humans [40]. It has been previously noted that *XRCC4*-patients have usually no clinical immunodeficiency, unlike in the other syndromes affecting the NHEJ system [15]. However, and as reported in some patients [7, 10, 11], we found quantitative abnormalities of B- and T-lymphocytes in P1 and P2, subclinical but possibly progressive. In contrast, *XRCC4* deficient mice showed a marked defective lymphogenesis with hypotrophic thymi, B and T-cell development arrest in early stages [38]. LIG4 residual activity in humans could provide a sufficient level of V(D)J coding segments recombination in B and T lymphocytes to ensure maturation of these immune cells despite subclinical defects in the diversity of immune repertoires [41]. Thus, the residual activity of LIG4 in context of complete XRCC4 deficiency could explain viability and the absence of clinical immunodeficiency in humans, due to a sufficient level of V(D)J recombination to maintain the diversity of the immune repertoire, but with MPD due to a lack of DSB repair in progenitor cells, leading to cycle cell arrest during embryogenesis.

### Functional investigations: towards standardized tests to reclassify VUS in XRCC4

Ionizing radiation is the main cause of DSBs [1], and patients with NHEJ defects logically presents an elevated cellular radiosensitivity, as demonstrated in vitro in *XRCC4* patients’ skin derived fibroblasts [7, 10, 12, 13, 40, 42]. Instead of skin fibroblasts, we reproduced these results for P1 and P2, carrying the known pathogenic p.(Trp43 Arg) variant in homozygous state [7, 9], with a survival assay on peripheral T lymphocytes, exposed to increasing doses of ionizing radiations (Fig. 3A). Moreover, although patients with biallelic pathogenic variants in *XRCC4* appear to be immunocompetent, 3/6 patients of our series show biological immune abnormalities, and subclinical but significant abnormalities in the diversification of the immune repertoire have been demonstrated previously, such as reduced competence for V(D)J recombination [7]. We reproduced this observation in P1 and P2 with new techniques: using flow cytometry, we showed that the proportion of TCR-Va7 expressing T-cells is decreased in non-MAIT T-lymphocytes (Fig. 3B), while multiplexed PCR and sequencing of coding segments (PROMIDISα) revealed a signature compatible with patients suffering from other V(D)J recombination defect (Fig. 3C). Together, these results corroborate a V(D)J recombination defect in *XRCC4*-patients. It is still not clear whether these anomalies have a clinical significance, but they could represent a useful biomarker for diagnosis. Indeed, while *XRCC4* loss-of-function variants are straightforward to interpret, new missense or splicing variants will require proof of their effect. The combination of these three tests demonstrating increased radiosensitivity and impaired V(D)J recombination will be of great help in reclassifying VUS. In particular, this PBMC-based approach has the significant advantage of rapid response (7 days), and is therefore less expensive than studies on fibroblasts which require several weeks of culture. This approach is also more acceptable, as a single blood sampling is required, without the need for invasive sampling such as skin biopsies.

## Conclusion

We present here clinical and molecular data for 7 new patients with *XRCC4*-related MPD, including the first antenatal diagnosis reported to our knowledge. This work extends the phenotype with novel features including transient increase in nuchal translucency, congenital glaucoma, oligodontia, hepatic steatosis, seizures, essential tremor, thumb abnormalities, café-au-lait and hypochromic macules, dermatofibrosarcoma, mandibular osteoid osteoma, and pancytopenia. We provide new evidence supporting tumor susceptibility in these patients, radiosensitivity, and subclinical but significant immune abnormalities. We recommend that these patients be carefully monitored for psychomotor development and nutrition in childhood, with screening for renal, sensory and urogenital abnormalities and neurological, endocrinological, cardiac and hematological monitoring adapted to the clinical and biological signs. Biological monitoring of blood count, glycaemia, liver and renal function tests, lipid and thyroid screening should be performed regularly, as well as immunophenotyping at less frequent intervals. We recommend avoiding exposure to ionising radiation in the context of medical care, and recommend special precautions regarding the use of genotoxic treatments in these patients. We also propose the combination of three PBMC-based functional assays, highlighting radiosensitivity and V(D)J recombination defect as a result of the NHEJ system impairment, that could contribute to the diagnosis of patients with VUS in *XRCC4*, using a fast, cost-effective and non-invasive approach. Together, these data will contribute to adapt the diagnosis, genetic counselling, follow-up and management of these patients.

## Supplementary information

Supplemental Material, Methods and Results

Supplemental Table 1

Supplemental Table 2

## Data Availability

The data that support the findings of this study are available upon request from the corresponding author. The data are not publicly available due to privacy or ethical restrictions. The newly identified p.(Glu13Ter) and p.(Arg205Ter) variants have been added as pathogenic in the ClinVar database (RCV004798185.1 and RCV004798186.1, respectively).
