# Supplementary material for: XRCC4-related microcephalic primordial dwarfism: description of a clinical series of 7 cases, phenotype expansion and new diagnostic approaches
Source: Eur J Hum Genet. 2025 Mar 20;33(7):842–51. doi: 10.1038/s41431-025-01821-0 (PMC12229645; doi:10.1038/s41431-025-01821-0)
Supplement: Supplementary file 1 — Supplemental Material, Methods and Results [file 41431_2025_1821_MOESM1_ESM.docx]

**Supplemental Material and Methods**

**Family 1**

Trio Exome Sequencing (ES) was performed and analyzed at the University Hospital of Lyon. Library preparation was performed with the Medexome kit (Roche) following manufacturers’ instructions. Paired-end 2x150 sequencing was performed on a NextSeq500 instrument (Illumina). Genomic alignment against the hg19/GRCh37 assembly and variant calling were, respectively, done with BWA-MEM v.0.7.12 (Li and Durbin, 2009) and GATK HaplotypeCaller v.3.4 (Broad Institute, Boston, MA, USA) while QC were evaluated using DeCovA (Dimassi et al., 2015). Only highly confident variants were kept for analysis (total depth >9; alternative allele depth >4; no strand bias; mosaicism >10%). Rare variants were considered as having a frequency below 1% in gnomAD database.

**Family 2**

Trio exome Sequencing was performed at the University of Edimburg and analyzed at Lille University Hospital. Library preparation, targeting exons and flanking introns (+/10pb), was performed with the SureSelect XTHSV2 kit (Agilent Technologies, Santa Clara, CA, USA). The indexed library was subjected to paired-end sequencing on a Hiseq400 (Illumina, San Diego, CA, USA). Sequence reads were then aligned to the human reference genome (GRCh38/hg38) and genetic variants were identified and annotated using Dragen (Illumina, San Diego, CA, USA) and in-house programs.

**Family 3**

Exome sequencing was performed in index-parent trio. Genomic DNA was captured using Agilent in-solution enrichment methodology (SureSelect) with biotinylated probes library (Human All Exon v5-50Mb, Agilent), followed by paired-end sequencing on Illumina HiSEQ 2000. The bioinfomatics analysis of sequencing data is based on the Illumina pipeline (CASAVA1.8.2) and was performed by IntegraGen (Evry, France). Sanger validation was performed to confirm the compound heterozygous variants in the *XRCC4* gene in proband, and the biparental inheritance in parents. Chromosomal breakage analysis after Mitomycine-C treatment on metaphasic chromosomes was performed following national protocol for Fanconi anemia diagnosis (1).

**Family 4**

DNA isolation from EDTA blood was carried out following standard protocols. Samples were subjected to a targeted multigene panel analysis of 119 microcephaly-associated genes. For library preparation, SureSelectTM QXT target enrichment kit (Agilent) with enzymatic fragmentation was used following manufacturer’s protocol (Agilent). Libraries were sequenced on an Illumina NextSeq 550 (Illumina, San Diego, CA), and Sequence Pilot (jsi medical systems GmbH) software was used to align sequences to a human reference genome (hg19) and for single nucleotide variant (SNV) calling. Parental samples were not available.

**Family 5**

Trio Exome Sequencing was performed using an adapted GATK-based approach and following a previously described protocol (2).

**Family 6**

Fetal growth was measured by ultrasound and assessed using the curves of Robinson et al. for crown-rump length (3) and Verburg et al. for head circumference, femur length, biparietal diameter and abdominal circumference (4). A prenatal Trio Exome Sequencing was performed on DNA extracted from chorion villus biopsy and parental blood samples, using a 4700 genes virtual panel for variant prioritization in multiple congenital anomalies, following a previously described protocol (5).

**Supplemental Results: clinical reports**

**Family 1**

The first patient (P1, **Figure 1A-C**), a 13-year-old boy, was the first of three siblings born to parents from eastern Europe, who are not known to be related, with no relevant family history. His antenatal history is unknown, but he was born at 39 weeks of gestation (WG), hypotrophic with a weight of 1500 g (-4 standard deviation SD), a height of 36 cm (-7.5 SD) and an occipitofrontal circumference (OFC) of 30 cm (-4 SD). His growth then proved steady, with a height and weight of around -5 SD, but his OFC gradually decreased. At age 10, he weighed 13.9 kg (-5.5 SD), measured 107 cm (-5 SD) and had an OFC of 43 cm (-7 SD). Facial features included sloping forehead, deep set eyes, low-set, posteriorly rotated ears with hypoplastic lobes, beaked nose with sharp nasal root and broad tip, low columella, short philtrum, and a pointed chin giving a triangular face shape. He has clinodactyly of the 5th fingers. He has undergone surgery for bilateral cryptorchidism. He also presented a dermatofibrosarcoma protuberans (Darrier-Ferrand sarcoma) of the left foot at 5 years, treated by surgical resection with poor healing. He had several bouts of bronchitis, ear infections and gastro-enteritis, as well as episodes of asthma that required treatment. He had teeth extraction because of cavities, but no abnormalities in the shape of his teeth have been reported. His hair and nails are normal. Early motor acquisitions were considered normal: he walked at 14 months and could ride a bicycle at 5 years of age. Language was delayed, with first words appearing before 20 months. At 11 years, he said only a few words without associating them. Reading and writing were not acquired. Neurological examination reported brisk reflexes since 3 years old, and action tremor since the age of 10 years, impairing fine motor skills. At 12 years, polykinetic reflexes was reported in all four limbs, with bilateral ankle clonus, but no reported muscle weakness. Brain MRI was normal. At the last examination, at the age of 13, weight was 16.8 kg (-5 SD), height was 121.5 cm (-6 SD) and OFC was 43.5 cm (-8 SD). It was noted that the child walked on tiptoes with hollow feet. In addition, pigmentary anomalies with hyperpigmented areas located around the scar on the foot, but also on the lower limbs and the front of the wrists were noted for the first time. He has no hearing impairment.

The second patient (P2, **Figure 1D-F**), young sister of P1, presented with an intra-uterine growth retardation (IUGR) at 28 WG observed on antenatal ultrasound. She was born at 35+3 WG, hypotrophic with a weight of 1280g (-3.3 SD), a height of 36 cm (-6 SD) and an OFC of 26.5 cm (-4.5 SD). Her statural growth was regular, but she showed post-natal OFC inflection. At age 9, she weighed 9.5 kg (-5 SD), measured 98 cm (-6.3 SD) and had an OFC of 40 cm (-8 SD). The facial features were similar to her brother and was additionally noted broad thumbs with distal implantation. Early motor development was normal, with walking acquired at 12 months, but she presented language delay, with first words pronounced at 3 years and a few words without association nor sentences at 10 years. Neurological examination showed brisk and slightly diffused reflexes in the lower limbs since 3 years of age. At the last examination, at 12, weight was 11.5 kg (-6 SD), height was 104 cm (-7.5 SD) and OFC was 40.2 cm (-10 SD).

Because of their cognitive difficulties and low level of autonomy, both siblings attend school in a medicalized educational institute. For both, cardiac, abdominal and brain imaging (MRI for patient 1 and scanner in patient 2) revealed no anatomical abnormalities apart from the known microcephaly. Blood count was overall normal, apart from discrete lymphopenia in P1 and P2 at 6 and 5 years respectively. Despite the absence of recurrent infections, a mild lymphopenia was observed in P1 and P2 (1.38 and 1.37 giga/L at 6 and 5 years respectively [normal values for age (N): 2-8]), prompting immunophenotyping. Both had mainly CD4+ T-cell lymphopenia (446 and 528/µL at 6 and 5 years respectively [N: 700-2200], then 365 and 288/µL at 13 and 12 years [N: 530-1300]) with a particularly marked deficit in the naive CD4+ CD45RA+ subpopulation, as well as B-cell lymphopenia (146 and 443/µL at 6 and 5 years respectively [N: 390-1400], then 62 and 24/µL at 13 and 12 years [N: 110-570]). CD8+ and NK cells count was normal. A slight weight deficit in immunoglobulins was observed only in P2 at the age of 12 (IgG 5.99 g/L [N: 7.10-15.60], IgA 0.71 g/L [N: 0.65-3.56], IgM g/L 0.48 [N: 0.66-2.50]). Thus, this immune deficiency, although subclinical, appears to be progressive in P2, but not in P1 (**Table S2**). IGF-1, IGFBP3, and TSH/T4L were normal for both. P2 showed mild dyslipidemia and prediabetes at 12 years. Array-CGH and karyotype were normal in P2, with no increased chromosomal breakage after in vitro mitomycin-C treatment. Analysis of a microcephaly NGS panel was performed in 2016 in P1, but did not find any explanatory cause since *XRCC4* was not yet included among the 36 genes studied. The diagnosis was made 2021 by quatuor ES analysis, revealing in both patients a homozygous pathogenic variant in *XRCC4*: c.127T>C p.(Trp43Arg), present in each parent at the heterozygous state.

**Family 2**

P3 **(Figure 1H-I)** is a female, third child of non-consanguineous and healthy parents of European origin without relevant familial history. Pregnancy was marked by early proportionate IUGR at 18 WG. Amniotic fluid sampling was performed and showed a normal standard karyotype 46,XX. Delivery was provoked at 32 WG because of growth stagnation. Anatomopathology of the placenta was normal. Birth weight was 1180 g (-2.5 SD), height 37cm (-2.5 SD) and OFC 24 cm (-4 SD), Apgar score 10/10. She presented with respiratory failure requiring non-invasive ventilation the first week. Hearing tests, cardiac, abdominal and renal ultrasounds were normal. At 3 months of age, bilateral glaucoma was diagnosed and surgically repaired. Because of failure to thrive, a gastric tube was placed. She also had gastroesophageal reflux disease, treated by esomeprazole. She presented with global delay in psychomotor acquisitions, sitting at 16 months and saying two words at 2 years. Neurological examination showed peripheral hypertonia and stereotypical hand wringing. Brain MRI performed at 4 months was normal. She received specialized education and intensive re-education. From the age of 3, she experienced progressive pancytopenia, without adenopathy or hepatosplenomegaly. The blood count at 3 years 6 months showed aregenerative macrocytic anaemia with 9.3 g/dL of haemoglobin (normal range for age: 11.5-12.5), 2.610 G/L erythrocytes (N: 3.9-5.3), a mean corpuscular volume of 104 fL (N: 75-87), and 78 G/L reticulocytes (N: 20-120). A moderate thrombopenia was associated with 55 G/L platelets (N: 150-400), and a mild leukopenia with 3.38 G/L leukocytes (N: 5.5-15.5) and 0.9 G/L lymphocytes (N: 3.0-10.5), while neutrophil count was normal. B12 and B9 levels were normal. Immunophenotyping was not performed. Peripheral blood smear examination showed dacrocytes, suggestive of extramedullar dyshaematopoiesis as seen in myelofibrosis. However, the myelogram initially showed a bone marrow of normal appearance and cell abundance, with no abnormalities suggestive of myelodysplastic syndrome. She was first treated symptomatically with folic acid, then pancytopenia progressively worsened towards bone marrow aplasia, and required iterative red blood cells transfusions every month, with preventive cotrimoxazole as anti-infective treatment. At 4 years, she presented with two episodes suggestive of generalized convulsive seizure, tonic-clonic then tonic, with loss of consciousness in both episodes. The electroencephalogram, performed on the day of the second episode, showed polyspike-waves and slow waves, predominantly bi-frontal, increased during sleep. A treatment with Levetiracetam was introduced, followed by Vigabatrin because of haematological disorders, and she no longer had seizures. At 5 years, walking was not achieved, she only produced few words and was still fed with gastric tube with almost no oral feeding. She showed MPD with a weight of 8,18 kg (-7 SD), height 80 cm (-7 SD) and OFC 37.5 cm (-9 SD). Morphological features included micrognathia, prominent nasal bridge and small teeth. She died at 5 in a context of fever and hemoptysis, with no identified triggering factor. Autopsy was declined by the family. Trio ES revealed pathogenic compound heterozygous biallelic variants in *XRCC4* gene, the c.25delC variant resulting in the p.(His9ThrfsTer8) frameshift inherited from the mother and the c.482G>A p.(Arg161Gln) missense variant inherited from the father.

**Family 3**

The fourth patient (P4, **Figure 1J-Q**) is a 22-year-old male, third child of unrelated parents, with no family history. During pregnancy, ultrasounds at 12 WG revealed IUGR and suspected left renal agenesis. At birth, after labor induction at 37 WG, the parameters were below the normal range for gestational age with a weight of 2200 g (-2.1 SD), length of 44 cm (-2.2 SD) and OFC of 28.5 cm (-4 SD). The neonatal period was marked by feeding difficulties, requiring nasogastric tube at 4 months, then gastrostomy tube during 2 years. Abdominal ultrasounds confirmed a left renal hypoplasia with normal renal function. Cardiac ultrasounds were normal. Skeletal radiographs showed delayed bone age with slender long bones. Brain MRI showed a slight lateral ventricular dilatation and pituitary hypoplasia with a relatively frontal lobe atrophy at 4-year-old. The patient presented repeated episodes of hypothermia that required multiple hospitalizations during infancy, evocative of impaired thermoregulation. He also had bilateral cryptorchidism, operated during childhood. Dental examination revealed a delay in the primary dentition loss and oligodontia with eight missing teeth. He also presented with astigmatism and hyperopia. At 12-year-old, the patient developed type 2 diabetes, currently stabilized with Metformine treatment. A hypogonadotropic hypogonadism was diagnosed at 16 years old. Excision of a mandibular ostoid osteoma was performed aged 20. Biological testings at 19 years old revealed hypertriglyceridemia (3.81 mmol/L [N: 0.60 – 1.70]) and dyscholesterolemia (total cholesterol 4.92 mmol/L [N: 3.9 – 5.7]; HDL 0.90 mmol/L [N: 1.0 – 2.0]; VLDL 1.73 mmol/L [N: 0.25 – 0.78]; LDL 2.29 mmol/L [N: 1 – 4.15]). Dyslipidemia rapidly worsened at 21 years: serum was lactescent, triglyceride reached a maximum value of 10.07 mmol/L, with hypercholesterolemia (total cholesterol 6.44 mmol/L; HDL 0.72 mmol/L; VLDL 2.01 mmol/L; LDL 3.16 mmol/L). It was complicated by a progressive hepatic steatosis, diagnosed on abdominal ultrasounds, without hepatic fibrosis (hepatic elastography by FibroScan of 6.3 KPa for a standard of 3.1 to 6.9 Kpa) and liver function tests were normal, with gamma-GT and transaminases within normal ranges. The patient benefited from combined therapy with Atorvastatin, Ezetimibe and Dapagliflozin since the age of 21 and his lipid profile is currently normalized at 23 (triglycerides 1.59 mmol/L; total cholesterol 2.60 mmol/L; HDL 0.97 mmol/L; VLDL 0.72 mmol/L; LDL 0.91 mmol/L). Regarding his neurodevelopment, early milestones were normal with first words at 15 months and good interactions, contrasting with the severe microcephaly. However, from 7 years of age, he had some learning difficulties, dyspraxia and dyslexia, that required specific education. In adulthood, a disabled worker status was obtained. On last clinical examination, aged 22, weight was at 37 kg (-3.96 SD), height at 146 cm (-4.87 SD), and OFC at 47.5 cm (-7.5 SD). Dermatological inspection revealed some pigmented and depigmented cutaneous spots macules on the skin. No abnormalities of the blood count were observed in this patient. Karyotype and array-CGH were normal, 46,XY. Interestingly, adjunction of Mitomycine-C on metaphase chromosomes showed a significant increase in chromosome breaks number (on 24% of metaphases), while this number was normal in spontaneous conditions. No radial pattern nor chromosomal rearrangement was observed. Trio ES revealed compound heterozygous pathogenic variants in *XRCC4*: c.25delC p.(His9ThrfsTer8) inherited from the mother and c.823C>T p.(Arg275Ter), inherited from the father.

**Family 4**

Patient P5 **(Figure 1R-X)** is a daughter from a family with no known medical history nor known consanguinity. The pregnancy was marked by oligohydramnios, and low weight for gestational age, but there was no pregnancy follow-up. She was born at 34 WG with a weight of 1425 g (-1.95 SD), a height of 37 cm (-2.97 SD) and an OFC of 28 cm (-2.18 SD). She had severe bilateral pes equinovarus with short Achilles tendons, probably secondary to oligohydramnios, and was treated with foot abduction orthosis. Hypotonia was noted as early as 6 months of age, followed by a global developmental delay: she pronounced her first words at 19 months and her first sentences after 2 years. She was sitting at 6 months but did not walk until 2.5 years-old, although the pes equinovarus probably hampered motor acquisitions. She had intellectual disability, stereotypic hand wringing, needed routines and help on all activities of everyday life. She presented severe feeding difficulties since birth, requiring nasogastric feeding until 7 months of age, then gastrostomy-tube feeding. At 11 years, she could drink some water but no oral feeding was possible. Regarding initial investigations, brain imaging, abdominal and cardiac ultrasound revealed no abnormalities. At 18 months, radiographs showed osteopenia with a severely delayed bone age. A biological assessment at age 9 revealed prediabetes with hyperinsulinemia. At age 11, she weighed 17.9 kg (-4.5 SD), was 105.5 cm tall (-6.7 SD), had an OFC of 39 cm (-10 SD). A hypergonadotropic hypogonadism was diagnosed, (FSH 71.3 mIU/ml [N: 2.1-11.1], LH 41.3 mIU/ml [N <11.9 mIU/ml], Estradiol 26 pg/ml [N: 30-400]), associated with hypertriglycidemia (triglyceride 2.57 g/L [N <1.5 g/L], total cholesterol 1.65 g/L [N <1.90 g/L], HDL cholesterol 0.45 g/L [N > 0.48 g/L], LDL cholesterol 0.98 g/L [N < 1.16 g/L]). Clinical examination showed low set ears without superior crus of antihelix, thin upper lip, short palpebral fissures, brachymesophalangy of the fifth finger, acanthosis nigricans on the neck and armpits, and hyperhydrosis of the hands. Karyotype and array-CGH were normal, 46,XX. A multigene panel sequencing revealed two pathogenic truncating variants in *XRCC4*, c.25delC p.(His9ThrfsTer8) and c.673C>T p.(Arg225Ter). Segregation study was not possible due to the impossibility to obtain parental blood samples.

**Family 5**

Patient P6 is a 11-month boy, born to unrelated parents with no family history. During pregnancy, a harmonious IUGR was noted at 20 WG. Labour was induced at 37+3 WG due to ongoing concerns with his growth. He was born healthy, with a weight of 2230g (-2.5 SD), a height of 48 cm (-0.5 SD), and an OFC of 30.5 cm (-2.5 SD). A sacral dimple was noted with borderline low termination of the spinal cord at spinal ultrasound, with no indication for surgery. Growth retardation became more pronounced and at 11 months, he weighed 5.8 kg (-3.8 SD), was 67 cm tall (-3.7 SD) and had an OFC of 40.4 cm (-4.4 SD). He has mild global developmental delay with predominant difficulties in expressive speech, and at 4 years he still cannot count beyond 3. Biological investigations, including full blood count, thyroid, renal and liver function tests, CKs, glucose, lactate, acylcarnitine profile, homocysteine and mucopolysaccharidosis screen, were all normal. Immunophenotyping was not performed. Brain MRI showed no abnormalities, and genetic testing for Silver-Russel syndrome was negative. Trio ES revealed two pathogenic compound heterozygous variants in *XRCC4* (NM_003401): the c.673C>T p.(Arg225Ter) inherited from the father, and c.37G>T p.(Glu13Ter) inherited from the mother.

**Family 6**

Patient P7 was a male fetus, fifth pregnancy from unrelated parents. They already had two healthy children and 2 early miscarriages (at 7 and 8.5 WG respectively). This pregnancy resulted from ovulation induction due to polycystic ovary syndrome. There was no other relevant family history. At 12+2 WG, the fetus had an increased nuchal translucency of 4.9 mm, a growth restriction around -3.3 SD for crown-rump length (48.6 mm) and -5 SD for head circumference (50.5 mm). A week later, the nuchal translucency was normalized (1.1 mm) but growth was still delayed. Non-invasive prenatal testing for aneuploidy was normal. At 14 WG, the proportionate early IUGR was confirmed. The fetus had a crown-rump length of 63.1 mm (-4.2 SD) and a head circumference of 69.4 mm (-6 SD). Biparietal diameter (19.3 mm), abdominal circumference (58.2 mm) and femoral length (8.9 mm) were also <-2.3 SD. A chorion villus biopsy was done. QF-PCR and SNP-array on fetal DNA were normal. Because of the recurrent miscarriages, chromosomal analysis was performed for parents and showed normal karyotypes. Trio ES identified pathogenic compound heterozygous mutations in *XRCC4* (NM_003401): c.25delC p.(His9Thrfs*8) inherited from the mother and c.613C>T p.(Arg205*), inherited from the father. Pregnancy was terminated at 16+2 WG and autopsy was declined by the parents.

**Supplemental Table S1** : *See Excel Sheet*
Detailed clinical and molecular data for patients newly and previously described.

**Supplemental Table S2** : *See Excel Sheet*

Immunological abnormalities in patients P1 and P2.
Values below the minimum threshold are shown in bold.

**Supplemental Figure 1**: T cell Activation proliferation as determined by cell counts at 6 days of culture. Results are expressed the proliferation relative to a healthy control sample.

**
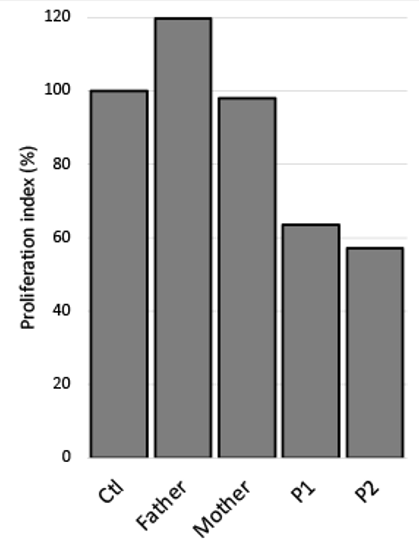
**

**Web Resources**

GnomAD v4.0.0 : https://gnomad.broadinstitute.org/

CADDv1.6 : https://cadd.gs.washington.edu/

ProteinPaint : https://proteinpaint.stjude.org/

**Supplemental References**

1. Castella M, Pujol R, Callén E, Ramírez MJ, Casado JA, Talavera M, et al. Chromosome fragility in patients with Fanconi anaemia: diagnostic implications and clinical impact. J Med Genet. 1 avr 2011;48(4):242‑50.

2. Knapp KM, Poke G, Jenkins D, Truter W, Bicknell LS. Expanding the phenotypic spectrum associated with DPF2: A new case report. Am J Med Genet A. 2019;179(8):1637‑41.

3. Robinson HP, Fleming JEE. A Critical Evaluation of Sonar “Crown-Rump Length” Measurements. BJOG Int J Obstet Gynaecol. 1975;82(9):702‑10.

4. Verburg BO, Steegers E a. P, De Ridder M, Snijders RJM, Smith E, Hofman A, et al. New charts for ultrasound dating of pregnancy and assessment of fetal growth: longitudinal data from a population-based cohort study. Ultrasound Obstet Gynecol. 2008;31(4):388‑96.

5. Corsten-Janssen N, Bouman K, Diphoorn JCD, Scheper AJ, Kinds R, el Mecky J, et al. A prospective study on rapid exome sequencing as a diagnostic test for multiple congenital anomalies on fetal ultrasound. Prenat Diagn. 2020;40(10):1300‑9.
